# The missed appointments and the tears of Niobe: the pain of not occurrence

**DOI:** 10.3402/ejpt.v4i0.22991

**Published:** 2013-10-30

## XIII ESTSS Conference: “Trauma and its clinical pathways: PTSD and beyond”, Bologna, June 2013 ORAL, JUNE 9 HALL LIZ

## Trauma e perdita

### Gli appuntamenti mancati e il pianto di Niobe: il dolore del non accadimento   11:00–11:15

#### Antonio De Luca Ministero della Giustizia, Cosenza, Italy

Quando qualcosa lacera il vissuto imponendosi come traumatico, esso espone altresì, come il negativo di un'antica foto, una prospettiva invisibile e di difficile comprensione: è il non accadimento di qualcos'altro. E’ l'appuntamento mancato dell'esistenza: ciò che avrebbe potuto verificarsi e non è accaduto. La morte improvvisa della persona cara non scava soltanto solchi profondi nelle pieghe dell'anima, a tratti invalicabili, non getta solo un'ombra acuta sulla via per l'accaduto, non pietrifica soltanto luoghi e battiti del tempo e della speranza, mentre tutto viene avvolto nell'area rarefatta della sospensione perchè impone altresì sequenze invisibili di ciò che non potrà più realizzarsi. E’ allora che compare il dolore del “non accadimento” insidiandosi quale carsica assenza di qualcuno e di qualcosa.

Della pietrificazione di Niobe solo il pianto scorga nel presente, in un tempo pietrificato anch'esso e diventa fonte dissetante per esistenze sospese tra i ricordi sulle macerie che non riescono a divenire rovine. Ciò che è sopravvissuto alla distruzione, nonostante tutto.

Quello che rimane nel vissuto dopo una distruzione non è soltanto il frammento degli accadimenti o il pungolo nella carne viva del momento traumatico, ma è anche ciò che avrebbe potuto essere e non è stato. Quale sofferenza allora per ciò che non è accaduto? Quale “sintomatologia” ed “elaborazione” di ciò che non è mai avvenuto?

Saranno discusse situazioni cliniche legate ad appuntamenti mancati, i vissuti emersi e l'aiuto terapeutico possibile.

**Bibliografia**

De Luca, A. (2000). Gli appuntamenti mancati e lo sguardo di Sisifo. *Comprendre, 10*, 89–99.

De Luca, A. (2011). *Tra le rovine dell'esistenza. Sofferenza Psicoterapia Ripresa* (Intr. di B. Callieri, Pres. di G. Di Petta). Roma: Edizioni Universitarie Romane.

De Luca, A. (in press). *La nostalgia infinita dell’altro e di sé: gli appuntamenti mancati e il dolore del non accadimento*, in A. De Luca, A. M. Pezzella (edd.), *Con i tuoi occhi. Sull'intersoggettività*. Milano: Mimesis.

### The missed appointments and the tears of Niobe: the pain of not occurrence

When something breaks the lived imposing itself as traumatic, it also exposes a prospect invisible: it is the not the occurrence of something else. It is the existential missed appointment: what would have happened but did not happen. Suspended along the history, the sudden death of the beloved person not only digs deep furrows in the folds of the soul. It does not throws only a shadow acute on track for what happened, it don't petrify places and the beat of time and hope while everything is wrapped in the area of rarefied suspension. It also requires invisible sequences of what, from that moment onwards, cannot be achieved together. All those moments are important, both in daily life and in the uniqueness of an event. It is then that appears as the pain of not occurrence, laying a trap for karst of absence of someone/something. In the petrification of Niobe, only tears appear in a frozen time being also source capable of quenching thirst lives suspended between the memories on the pile of rubble not becoming ruins, in spite of all. What remains after an emotional destruction is that fragment of the events together with what didn't happened. What suffering then for what didn't happen? Which “symptoms” and “processing” of that never happened? Will be discussed clinical situations and affects related to “missed appointments” and the possible therapy.

